# The Role of Intact and Disintegrated Egg Yolk Low-Density Lipoproteins during Sponge Cake Making and Their Impact on Starch and Protein Mediated Structure Setting

**DOI:** 10.3390/foods10010107

**Published:** 2021-01-06

**Authors:** Sarah C. Pycarelle, Geertrui M. Bosmans, Bram Pareyt, Kristof Brijs, Jan A. Delcour

**Affiliations:** 1 Laboratory of Food Chemistry and Biochemistry and Leuven Food Science and Nutrition Research Centre (LFoRCe), KU Leuven, Kasteelpark Arenberg 20, 3001 Heverlee, Belgium; kristof.brijs@kuleuven.be (K.B.); jan.delcour@kuleuven.be (J.A.D.); 2 Puratos NV, Industrialaan 25, 1702 Groot-Bijgaarden, Belgium; gbosmans@puratos.com (G.M.B.); bpareyt@puratos.com (B.P.)

**Keywords:** egg yolk lipids, sponge cake, low-density lipoproteins, lipoprotein disintegration, gas cell stability, cake quality, cake structure setting

## Abstract

The main sponge cake ingredients are flour, sucrose, eggs and leavening agents. Exogenous lipids (e.g., monoacylglycerols) are often used to increase air–liquid interface stability in the batter. There is a consumer trend to avoid foods containing such additives. We here reasoned that egg yolk may be an alternative source of surface-active lipids and set out to study the role of egg yolk lipids during sponge cake making. This was done by relocating or removing them prior to batter preparation using ethanol treatments and examining how this affects cake (batter) properties and structure setting during baking. Most egg yolk lipids occur within spherical low-density lipoproteins (LDLs) which were disintegrated by the ethanol treatments. Results showed that egg yolk lipids impact air–liquid interface stability and less so cake structure setting. To prepare high-quality sponge cakes by multistage mixing preferably intact LDLs or, alternatively, their components are needed to incorporate sufficient air during mixing and to stabilize it after mixing. It was also shown that the batter contains intact LDLs in the continuous phase and disintegrated LDLs at air–liquid interfaces. Sponge cake contains intact LDLs in the cake matrix, disintegrated LDLs at air–crumb interfaces and disintegrated LDLs incorporated into the protein network.

## 1. Introduction

Sponge cakes are typically prepared from flour, sugar (i.e., mostly sucrose), eggs or egg fractions, leavening agents and optionally exogenous lipids [1,2,3,4]. The lipids in their recipe stem from flour, eggs and optionally from lipid-like additives. Egg white contains negligible amounts of lipids, while approximately two-thirds of egg yolk dry matter consists of lipids [5]. The latter consist of ca. 66% triacylglycerols, 28% phospholipids, 5% cholesterol and 1% other lipids [6,7]. The main yolk phospholipids are phosphatidylcholines (73%), phosphatidylethanolamines (16%), lysophosphatidylcholines (6%), sphingomyelin (3%) and lysophosphatidylethanolamines (2%) [8]. Exogenous lipids used for sponge cake making are typically monoacylglycerols and polyglycerol esters of fatty acids. They are very efficient in stabilizing air–liquid interfaces [3,9,10]. Their use facilitates the incorporation of many gas cells into the batter during mixing and also increases gas cell stability after mixing. Many stable gas cells eventually lead to high-quality sponge cakes with high volume and soft texture [2,4,11]. However, recently, consumers have been perceiving these additives negatively [12] and have been looking for food without or with less additives (i.e., clean label food products).

Most egg yolk lipids naturally occur within low-density lipoproteins. These micelle-like structures have a core of nonpolar lipids (i.e., triacylglycerols and cholesterol esters) surrounded by phospholipids, cholesterol and apoproteins. They are surface-active and can stabilize oil–liquid and air–liquid interfaces by spreading out and distributing their components along such interfaces [13,14,15,16].

Many years ago, the role of egg yolk in sponge cake making was studied by Kamat et al. [17] and Graham et al. [18]. Kamat et al. [17] prepared sponge cakes from recipes in which egg yolk was replaced by egg yolk plasma and/or granules. They suggested that low-density lipoproteins assist aeration during mixing and that their functionality relies on their integrity. Although these hypotheses are plausible, they were based on subjective evaluations rather than on quantitative measurements of batter densities or lipoprotein integrity. Against this background, part of this study aimed at confirming these hypotheses and further elaborating on the importance of low-density lipoproteins at the air–liquid interface during mixing but also during early baking.

Graham et al. [18] revealed that the integrity of low-density lipoproteins is lost upon setting of the cake structure during baking. They used hexane-extractable lipid content as a measure of the disintegration of low-density lipoproteins during heating [18]. This raises the question whether the released lipid fraction impacts timing and the extent of sponge cake structure setting during baking. Cake batter transforms into a solid cake because of two gel-forming phenomena: starch gelatinization and protein polymerization [19].

Recent literature has mostly focused on the role of egg yolk plasma and egg yolk granules in batter-type cakes [20,21,22]. As such systems typically contain margarine, oil or shortening, egg yolk lipids or low-density lipoproteins in such cases cannot only adsorb at air–liquid but also at oil–liquid interfaces as mentioned above. Furthermore, lipid crystals when present in the system also adsorb at the air–liquid interface during mixing [19] and may impact egg yolk lipid functionality at that interface. As a result, insights into egg yolk lipid functionality in batter-type cakes are not applicable to foam-type cakes.

Against the above background, the present study aimed at developing a holistic view on the role of egg yolk lipids during the entire sponge cake making process. The following research questions were defined.

- To what extent do egg yolk lipids determine sponge cake **batter properties**? How can the resultant insights be related to their role at the air–liquid interface during mixing?
- To what extent do egg yolk lipids determine sponge **cake properties**? How can the resultant insights be related to their role at the air–liquid interface during early baking?
- Do egg yolk lipids impact sponge cake (batter) properties by impacting timing and the extent of sponge **cake structure setting** during late baking?

To answer these questions, sponge cakes were prepared with either standard egg yolk, egg yolk containing relocated lipids or defatted egg yolk. Because low-density lipoproteins were expected to be disintegrated in egg yolk containing relocated lipids, lipoprotein integrity was monitored by determining hexane-extractable lipid contents of the abovementioned batter and cake samples.

Sponge cake batters were prepared using a traditional multistage mixing method in which egg white and egg yolk were whipped separately from each other, each with part of the sugar. Afterwards, the flour was mechanically added.

The batter properties that were evaluated were density and apparent viscosity. Batter density depends on the amount of air incorporated during the entire mixing procedure, while its viscosity is related to gas cell stability. Indeed, a higher batter viscosity minimizes drainage, gas cell coalescence and bubble rise and hence results in a higher gas cell stability [23]. The cake properties evaluated were cake volume and crumb hardness, cohesiveness, springiness and structure. Cake structure setting was investigated by monitoring the time- and temperature-dependence of starch gelatinization and protein denaturation by differential scanning calorimetry (DSC) and of protein polymerization by size-exclusion high-performance liquid chromatography (SE-HPLC).

To the best of our knowledge, this is the first thorough examination of the role of egg yolk lipids during the different stages of foam-type cake making in general and during baking in particular. The results clearly showed that egg yolk lipids impact gas cell incorporation and stability but not the timing and extent of cake structure setting. In addition, the presence of intact and disintegrated low-density lipoprotein populations in batter and cake was established. The insights gained here are a valuable starting point for developing clean label foam-type cakes.

## 2. Materials and Methods

### 2.1. Ingredients and Solvents

Wheat flour (Halm flour, 14.0% moisture content and 10.4% protein content), rice starch (10.0% moisture content) and leavening agents (sodium acid pyrophosphate (number 15) and sodium bicarbonate) were from Paniflower (Merksem, Belgium), Beneo (Wijgmaal, Belgium) and Budenheim (Budenheim, Germany), respectively. Eggs were purchased locally, stored at 3 °C and used before their “best before” date. High performance liquid chromatography grade hexane was from Thermo Fischer Scientific (Geel, Belgium) and ethanol (Disinfectol, denatured with 3% diethyl ether) from Chem-Lab (Zedelgem, Belgium). Other chemicals were obtained from Sigma-Aldrich (Bornem, Belgium) and of analytical grade unless indicated otherwise.

### 2.2. Analysis of Moisture and Protein Contents

Moisture contents of flour and rice starch were determined according to AACC method 44-15.02 [24]. Protein contents of flour and egg yolk pellets (see Section 2.3) were determined based on AOAC method 990.03 [25]. For this, approximately 500 mg flour or 90 mg egg yolk pellet was accurately weighed and analyzed with a VarioMax Cube N (Elementar, Hanau, Germany). Nitrogen-to-protein conversion factors were 5.7 for flour and 6.25 for egg yolk pellets. All analyses were performed in triplicate.

### 2.3. Extraction of Lipids from Freeze-Dried Egg Yolk with Ethanol

Fresh eggs were separated into egg white and egg yolk as commonly done. Both fractions were frozen with liquid nitrogen and stored at −20 °C until freeze-dried. The ice condenser temperature was −55 °C. Fully dried egg white and egg yolk contained 1.0 to 5.0% moisture. Both were ground with mortar and pestle and stored at 7 °C until further use. In what follows, fresh egg white and egg yolk are further referred to as EW_F_ and EY_F_, while their freeze-dried versions are denoted as EW_FD_ and EY_FD_.

**Figure 1:**
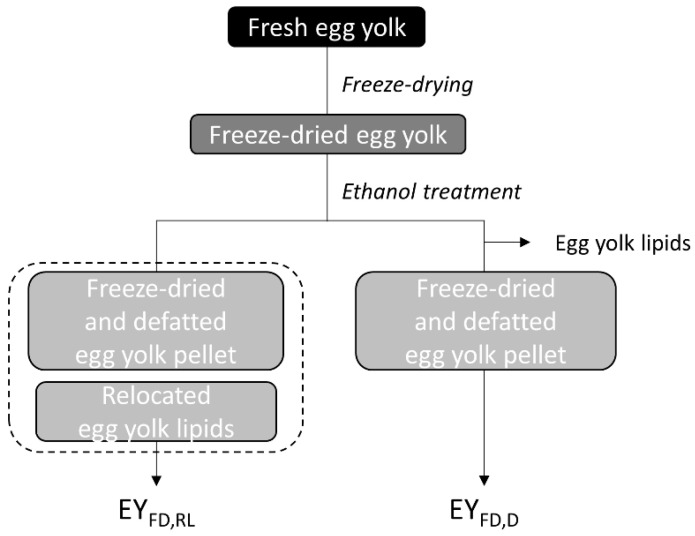
Fresh egg yolk was freeze-dried after which it was treated with ethanol. Egg yolk lipids were either relocated (EY_FD,RL_) or removed (i.e., defatted egg yolk, EY_FD,D_).

Ethanol was chosen to extract lipids from EY_FD_ since it extracts almost all egg yolk lipids and only a limited portion of protein, and because it is easily evaporated. To extract egg yolk lipids (Figure 1), six 250 g EY_FD_ dry matter portions were shaken in 1250 mL ethanol (60 min, 150 rotations/min, room temperature). Next, the ethanol phases were removed by Büchner filtration. A second batch of 1250 mL ethanol was added to the residues and the procedure was repeated. Supernatants were pooled per portion of EY_FD_ and the solvent was removed at 30 to 35 °C by rotary evaporation (Rotavapor R-3000, Büchi Labortechnik, Hendrik-Ido-Ambacht, The Netherlands). The obtained lipids were weighed and stored at −80 °C under nitrogen atmosphere until used. The resultant pellets were spread on filter paper and dried overnight under a fume hood. The next day, they were ground with a laboratory mill (model A10, IKA, Staufen, Germany), sieved (mesh size 400 µm) and stored at 7 °C until further use.

The combination of comminuted egg yolk pellets and the lipids extracted therefrom is further referred to as egg yolk containing relocated lipids (EY_FD,RL_) (Figure 1). Defatted egg yolk (EY_FD,D_) was obtained by drying egg yolk pellets (as above) after first discarding the supernatants containing the extracted lipids (Figure 1).

### 2.4. Batter Preparation and Cake Baking

As freeze-drying was an inherent part of the experimental approach used here, the impact thereof on egg white and egg yolk functionality during sponge cake making was studied first. Prior to batter preparation, EW_FD_ and/or EY_FD_ were hydrated to the moisture content of their fresh counterparts by suspending them in deionized water and stirring overnight at 7 °C in a glass beaker covered with Parafilm M (Bemis, Neenah, WI, USA). When preparing sponge cake batter with EY_FD,RL_ or EY_FD,D_, the egg yolk pellet with known protein content (see Section 2.2) was dosed such that the same amount of egg yolk protein as that in batter prepared with EY_F_ was included. Furthermore, it is of note that the recipe for EY_FD,D_ batter was not adapted to compensate for the removed egg yolk lipids. The water level in each recipe was adapted to obtain a batter moisture content of 35.8%. Egg yolk pellets were also hydrated as above prior to batter preparation.

The sponge cake recipe contained flour (281.0 g), sucrose (247.0 g), EW_F_ (176.0 g), EY_F_ (64.0 g), deionized water (93.6 g), rice starch (30.8 g), sodium acid pyrophosphate (6.6 g) and sodium bicarbonate (4.8 g). All sponge cake batters were prepared according to a multistage mixing method developed at our group [26]. How this method was applied to prepare sponge cakes with treated egg yolk samples (see Section 2.3) is further clarified below.

When EY_FD,RL_ or EY_FD,D_ were used to prepare sponge cake batter, the first step consisted of whipping hydrated EW_FD_ together with 1/3rd of the total recipe sucrose. To prepare sponge cake batter with EY_FD,RL_, the second step consisted of mixing the hydrated egg yolk pellet along with the extracted egg yolk lipid fraction (see Section 2.3), 2/3rds of the sucrose, water, 50% of the flour and rice starch. Based on the amount of lipids extracted (see Section 2.3), egg yolk lipids were dosed to obtain the lipid content present in EY_F_. To prepare sponge cake batter with EY_FD,D_, the second step consisted of mixing the hydrated egg yolk pellet, 2/3rds of the sucrose, water, 50% of the flour and rice starch. Finally, egg white and egg yolk mixtures were homogenized and the remaining flour was folded in mechanically.

From each of the six different batches (see Section 2.3), batter was prepared (Table 1). All batters were baked into cakes in a plate oven (30 min, top temperature 180 °C, bottom temperature 160 °C). The resultant cakes were then allowed to cool to room temperature. Details on this procedure are described elsewhere [26].

The abbreviations referring to the abovementioned batter/cake samples are introduced in Table 1. For instance, batter prepared with EW_FD_ and EY_FD,RL_ is denoted as EW_FD_/EY_FD,RL_ batter. The number of batters prepared per recipe are also shown.

### 2.5. Analysis of Batter and Cake Properties

The sponge cake batter properties analyzed in this study were its density and apparent viscosity. Cake properties included cake volume and crumb hardness, cohesiveness, springiness and structure. Hardness values (N) were corrected for cake density and reported as corrected hardness (N*cm^3^/g). Crumb structure was evaluated by calculating the average gas cell size (mm^2^) and the number of gas cells per cm^2^. The methods used are thoroughly described in Pycarelle et al. [26].

### 2.6. Analysis of Hexane-Extractable Lipid Content of Batter and Cake Samples

Batter and cake samples (see Section 2.4) were flash-frozen and freeze-dried. They were then ground with a mortar and pestle before lipid extraction. To extract lipids, triplicate samples (100.0 mg dry matter) suspended in hexane (5.0 mL) were shaken for 60 min (150 rotations/min, room temperature) and centrifuged (3000× *g*, 10 min, 23 °C). The pellets obtained after removing the supernatant were extracted once more as above. The solvent was removed from the pooled supernatants with a rotational vacuum concentrator (Martin Christ, Osterode am Harz, Germany, 800 Pa, 40 °C). The resulting hexane-extractable lipids were accurately weighed and their contents expressed as percentages of sample dry matter.

### 2.7. Analysis of the Timing and Extent of Starch Gelatinization and Protein Denaturation during Sponge Cake Baking

Online and offline DSC experiments were performed to analyze the impact of prior egg yolk lipid relocation or removal on starch gelatinization and protein (i.e., albumins and globulins from wheat flour and egg) denaturation during sponge cake baking [27]. EW_FD_/EY_FD_, EW_FD_/EY_FD,RL_ and EW_FD_/EY_FD,D_ batter/cake samples were analyzed together with an empty reference pan. Calibration was with indium. DSC analyses were with a Q1000 DSC (TA Instruments, New Castle, DE, USA) and data were processed with TA Instruments Universal Analysis software.

For each batch of batter, online DSC measurements were performed at least in triplicate. Here, the onset temperature of starch gelatinization and protein denaturation during sponge cake baking was measured in situ by applying a heating step similar to that during conventional sponge cake baking. For this, 5.0–10.0 mg fresh sponge cake batter was accurately weighed in aluminum DSC pans (Perkin-Elmer, Waltham, MA, USA). The pans were then hermetically sealed and heated from 0 to 98 °C at 7.3 °C/min.

Offline DSC measurements were also performed. Here, sponge cake baking was simulated by heating batter samples (200–300 mg in 2 mL Eppendorf tubes) in a water bath at 100 °C for different heating times (i.e., 30, 60 and 90 s and 2, 5, 10, 20 and 30 min). After each heating period batter/cake samples were frozen with liquid nitrogen, dried and ground. To avoid interference of lipid melting during DSC analyses, freeze-dried batter/cake samples were defatted with hexane prior to DSC analyses. Lipids were removed by suspending batter/cake samples (500 mg sample dry matter) in 5.0 mL hexane, shaking (150 rotations/min, 60 min, room temperature) and centrifugation (3000× *g*, 10 min, 23 °C). Afterwards, the supernatant was removed and the above extraction was repeated. The resulting pellet was dried under a fume hood overnight. These samples are further denoted as heated + defatted samples. For each recipe, these samples were prepared three times from individual sponge cake batters (Table 1).

For offline DSC analyses, deionized water was added (1:3 (*w*/*w*) sample dry matter:water) to accurately weighed heated + defatted samples (2.0–5.0 mg) in aluminum DSC pans. Pans were hermetically sealed and heated from 0 to 130 °C at 4 °C/min. DSC analysis was performed at least once for every heated + defatted sample. Endothermic enthalpies were quantified (ΔHs, expressed in J/g dry matter sample) and represent the contents of nongelatinized starch and nondenatured proteins as a function of baking time.

### 2.8. Analysis of the Timing and Extent of Protein Polymerization during Sponge Cake Baking

The impact of prior egg yolk lipid relocation or removal on time and temperature dependence of protein polymerization was monitored by determining the percentage of proteins extractable in sodium dodecyl sulfate (SDS) containing medium (i.e., 0.05 M sodium phosphate buffer containing 2.0% (*w*/*v*) SDS) (SDS EP) of the heated + defatted samples described above (see Section 2.7). The decrease in SDS EP values during baking reflects the formation of disulfide cross-links between proteins or thus the formation of a protein network [28].

For every heated + defatted sample proteins were extracted once with SDS containing medium. Total batter protein was extracted with SDS+DTT containing medium (i.e., SDS containing medium additionally containing 2.0% dithiothreitol (DTT)). The first extract is further denoted as SDS extract, while the second as SDS+DTT extract.

How proteins are extracted from sponge cake (batter) samples and how these extracts are then analyzed with SE-HPLC is thoroughly described elsewhere [4]. SDS EP values were calculated from SE-HPLC data according to Equation (1).

(1) $$\mathrm{SDS}\;\mathrm{EP}\;(\%)=\frac{\mathrm{total}\;\mathrm{area}\;\mathrm{SDS}\;\mathrm{extract}\;\mathrm{of}\;\mathrm{heated}+\mathrm{defatted}\;\mathrm{sample}\;}{\mathrm{total}\;\mathrm{area}\;\mathrm{SDS}+\mathrm{DTT}\;\mathrm{extract}\;\mathrm{of}\;\mathrm{defatted}\;\mathrm{sponge}\;\mathrm{cake}\;\mathrm{batter}\;}\times 100$$

### 2.9. Statistical Data Analysis

Data resulting from recipes that were processed more than once were submitted to statistical analysis by performing a one-way analysis of variance (ANOVA) after which a Tukey multiple comparison test was used to verify significant differences between mean values. These analyses were performed at a significance level (α) of 0.05 with JMP Pro 12 software (SAS Institute, Cary, NC, USA).

## 3. Results and Discussion

### 3.1. Impact of Prior Freeze-Drying of Egg White and Egg Yolk on Batter and Cake Properties

To unravel the role of egg yolk lipids during sponge cake making EW_FD_ and EY_FD_ were used. Hence, the impact of freezing, drying and grinding egg white and/or egg yolk prior to batter making was first investigated. For this, the properties of batters and cakes prepared with EW_F_/EY_F_, EW_FD_/EY_F_, EW_F_/EY_FD_ or EW_FD_/EY_FD_ were compared.

The use of **EW_FD_/EY_F_** instead of EW_F_/EY_F_ did not alter sponge cake batter density (Table 2). Hence, prior freezing, drying and grinding of egg white did not impact its foaming properties during batter preparation. It has earlier been observed that the sensory quality of meringues prepared with either EW_FD_ or EW_F_ is comparable [29]. Cake volume, crumb texture (i.e., corrected hardness, cohesiveness and springiness) and structure were also similar when using EW_FD_ instead of EW_F_ (Table 2). In contrast, **EW_F_/EY_FD_** batter had a higher density than EW_F_/EY_F_ batter (Table 2). The resulting EW_F_/EY_FD_ cakes were smaller and slightly harder than those prepared with EW_F_/EY_F_ while crumb cohesiveness and springiness values were comparable. Furthermore, crumb of the former cakes seemed to have smaller gas cells than that of the latter. Both crumb types contained similar numbers of gas cells per unit of area (Table 2). From the above, it is clear that freezing, drying and/or grinding negatively impacted egg yolk functionality during sponge cake making and especially during mixing. The use of **EW_FD_/EY_FD_** for sponge cake making negatively impacted batter and cake properties in a way similar to that observed when EW_F_/EY_FD_ was used (Table 2). Above, we already concluded that freezing, drying and grinding did not impact egg white functionality during sponge cake making. Hence, the negative impact of the use of EW_FD_/EY_FD_ rather than that of EW_F_/EY_F_ resulted from using EY_FD_ and not from using EW_FD_.

To understand why gas cell incorporation during batter making was reduced in the presence of EY_FD_ instead of EY_F_, one needs to consider low-density lipoproteins. These are the main components responsible for the surface-active properties of egg yolk. Low-density lipoproteins are spherical structures that make up ca. 68% of egg yolk dry matter [5,30] and are surface-active at air–liquid interfaces [15,30,31]. They contribute to aeration during mixing by first adsorbing and then spreading their components (i.e., triacylglycerols, phospholipids and apoproteins) along the air–liquid interface [5,15,17,18,30]. As aeration was impaired when using EY_FD_ rather than EY_F_ in cake making, it is very likely that at least part of the low-density lipoprotein population lost their surface-active properties when egg yolk was frozen with liquid nitrogen and dried.

Because proteins may experience denaturation at the protein–ice interface during freezing [32,33], it is reasonable to expect that freezing egg yolk denatured (part of) the apoproteins residing in the outer layer of low-density lipoproteins. Much of what occurs during heat-induced and freeze-thaw gelation of egg yolk, denaturation of apoproteins then likely destabilizes the spherical lipoprotein structure. Furthermore, when egg yolk is either heated or frozen and consecutively thawed, apoproteins denature and form an extended network through connections between lipoproteins [33,34,35]. Taken together, the changes in apoprotein secondary structure itself and/or the lipoprotein aggregation described above possibly lowered the surface-activity of low-density lipoproteins in EY_FD_. On top of that, apoproteins have been suggested to be responsible for lipoprotein anchoring at air–liquid interfaces [15]. Evidently, their denaturation may then also hamper lipoprotein adsorption at the air–liquid interface during the mixing of sponge cake batter.

**Figure 2:**
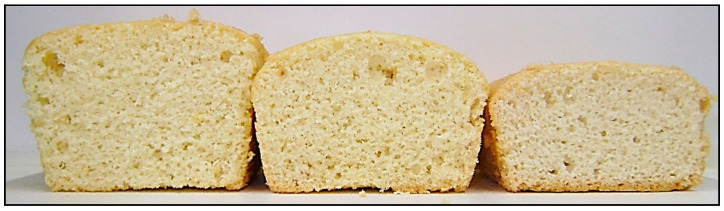
Sponge cakes prepared with (i) freeze-dried egg white and egg yolk (EW_FD_/EY_FD_, left), (ii) freeze-dried egg white and egg yolk with relocated lipids (EW_FD_/EY_FD,RL_, middle) and (iii) freeze-dried egg white and defatted egg yolk (EW_FD_/EY_FD,D_, right).

Irrespective of what caused EY_FD_ to be less functional than EY_F_ during sponge cake making, the use of EW_FD_/EY_FD_ did result in sponge cakes of an acceptable quality (Table 2 and Figure 2). This allowed us to use the recipe including EW_FD_ and EY_FD_ as reference to investigate the role of egg yolk lipids during sponge making (see Section 3.2 and Section 3.3).

### 3.2. Impact of Prior Relocation or Release of Egg Yolk Lipids on Batter and Cake Properties

Batter viscosity is determined by the water binding capacity of all batter constituents, the sucrose concentration in the batter [1] and by the amount of air cells incorporated during mixing [36]. The presence of more gas cells in the batter generally leads to a higher batter viscosity [4,26,37]. In the present case less air was incorporated in the EW_FD_/EY_FD,RL_ batter than in EW_FD_/EY_FD_ batter, while the former had a higher viscosity than the latter (Table 2). As sucrose levels were the same for both batters, the higher viscosity of EW_FD_/EY_FD,RL_ batter likely originated from an altered water binding capacity of batter constituents when using EY_FD,RL_ for batter preparation.

The undesired impact on batter density (and thus on air incorporation during mixing) was most likely caused by disintegration of low-density lipoproteins which itself was brought about by the ethanol treatment after freeze-drying (see Section 2.3). It is hypothesized that air incorporation during batter making was most likely impaired by the absence of intact surface-active low-density lipoproteins and/or by the presence of the lipid fraction released from disintegrated low-density lipoproteins. It has indeed been postulated early on that low-density lipoprotein integrity is important in the preparation of high-quality sponge cakes [17]. Furthermore, when low-density lipoproteins disintegrate, their nonpolar lipid core is released [18]. Previous work by our group [4] has shown that proteins dominate the air–liquid interface in sponge cake batter and form a coherent viscoelastic layer. However, not only proteins but also lipids can adsorb at the air–liquid interface in the batter [4]. In this case, the released egg yolk lipid fraction may adsorb at the interface where it hampers protein adsorption and/or protein–protein interactions, and thereby the formation of a viscoelastic protein layer. In other words, the presence of released egg yolk lipids likely limits gas cell stability in sponge cake batter. Recently, the same was suggested for the free lipid fraction in flour [26].

The volumes of the EW_FD_/EY_FD,RL_ cakes were acceptable but significantly lower (ca. 17%) than those of EW_FD_/EY_FD_ cakes (Table 2 and Figure 2). This along with the differences in their batter densities showed that the gas cells in EW_FD_/EY_FD,RL_ batter were quite stable after mixing and during early baking. The high viscosity of EW_FD_/EY_FD,RL_ (Table 2) likely contributed to gas cell stability because it limited coalescence, disproportionation and bubble rise [1,2]. The surface-active components released from low-density lipoproteins as a result of the ethanol treatment also probably stabilized the gas cells in the sponge cake batter to a certain degree.

Based on how egg yolk low-density lipoproteins stabilize air–liquid interfaces formed in a Langmuir trough apparatus [14,15], it is suggested that the different lipoprotein components (i.e., nonpolar lipids, phospholipids and apoprotein-lipid complexes) independently contributed to air–liquid interface stability in the batter. It has been shown that the surface layer made from triacylglycerols isolated from egg yolk collapses at lower surface pressure than layers made from phospholipids isolated from egg yolk [14]. Therefore, it is hypothesized that phospholipids released from lipoprotein structures are more likely to adsorb at air–liquid interfaces than released triacylglycerols. Next to phospholipids, apoproteins presumably also stabilize the air–liquid interface in the form of apoprotein-lipid complexes [15]. Whether such complexes still exist after the disintegration of low-density lipoproteins due to adsorption at the air–liquid interface or due to freeze-drying followed by an ethanol treatment remains unknown.

Cohesiveness and springiness values were significantly lower for EW_FD_/EY_FD,RL_ cakes than for EW_FD_/EY_FD_ cakes, whereas their corrected hardness values were similar (Table 2). As the crumb of the former contained larger gas cells than that of the latter, also less gas cells per unit crumb area were detected (Table 2).

From our results, it is clear that proteins and lipids when structured in low-density lipoproteins more efficiently stabilize the air–liquid interface during sponge cake making than when they occur separately. Hence, the integrity of these micelle-like structures is important for incorporating large numbers of gas cells during mixing and for obtaining sponge cakes with good texture and fine crumb. Furthermore, we carefully hypothesize that the spherical lipoprotein structure acts as a carrier for surface-active molecules and determines their structured diffusion to and adsorption at air–liquid interfaces in aqueous systems. In this view, the phospholipid monolayer in the outer layer of low-density lipoproteins is withheld after adsorption and immediately creates a stable “condensed” lipid layer. In contrast, when those phospholipids are not structured in spherical low-density lipoproteins (e.g., when using ethanol-treated egg yolk), they may still diffuse to the air–liquid interface but in a less structured way. When single (phospho)lipid molecules adsorb at the air–liquid interface, they form rather diffuse lipid layers wherein protein molecules easily perturb. Single lipid molecules also easily insert into protein layers formed at the interface [31]. The structural changes that low-density lipoproteins and their components undergo after adsorption at air–liquid interfaces is in our opinion an interesting topic to focus on in the future.

### 3.3. Impact of Prior Removal of Egg Yolk Lipids on Batter and Cake Properties

By removing egg yolk lipids from EY_FD_ by ethanol treatment (see Section 2.3), the protein content (expressed on dry matter base) increased from 33.4 ± 0.5% for EY_FD_ to 89.5 ± 2.4% for EY_FD,D_. Furthermore, the egg yolk pellets remaining after lipid extraction accounted for 34.7 ± 1.0% of the EY_FD_ dry matter which corresponded to about the protein content of EY_FD_. Thus, the solvent treatment was very effective as it removed approximately 95% of the lipids in EY_FD_.

The use of EW_FD_/EY_FD,D_ instead of EW_FD_/EY_FD_ for sponge cake making resulted in significantly higher batter viscosities and densities (Table 2). The latter were similar to those observed for EW_FD_/EY_FD,RL_ batter (Table 2). The negative impact on batter density likely resulted from having insufficient amounts of surface-active components during the second step of the multistage mixing method (i.e., mixing of EY_FD,D_, sucrose, water, rice starch and 50% of the flour, see Section 2.4). Indeed, it was visually observed that at that point in the process the mixture prepared with EY_FD,D_ was denser than those prepared with EY_FD_ or EY_FD,RL_. It is also likely that during the homogenization of the EY_FD,D_ mixture with the EW_FD_-sucrose foam (i.e., the third step of the mixing method, see Section 2.4) more air was lost than during the preparation of the EY_FD_ or EY_FD,RL_ batter. Because of the dense character of the EY_FD,D_ mixture, air incorporated into the EW_FD_-sucrose foam was probably pushed out during this mixing step.

Since EW_FD_/EY_FD,D_ cakes were smaller than EW_FD_/EY_FD,RL_ cakes even if their batters had comparable densities (Table 2 and Figure 2), more gas cells were lost during the baking of the former. Evidently, these results point to the importance of the separate components of low-density lipoproteins at the air–liquid interface during baking of EW_FD_/EY_FD,RL_ batter as thoroughly discussed in Section 3.2.

Cohesiveness and springiness readings of all sponge cake samples differed only slightly and the crumb structures of EW_FD_/EY_FD,D_ and EW_FD_/EY_FD,RL_ cakes were comparable (Table 2). EW_FD_/EY_FD,D_ cakes were almost bread-like (Figure 2) which was reflected by significantly higher corrected hardness values for the crumb of EW_FD_/EY_FD,D_ cakes than for that of the EW_FD_/EY_FD_ or EW_FD_/EY_FD,RL_ cakes (Table 2). Although cake hardness was corrected for its density, it did not correct for differences in cell wall structure. Hence, it is reasonable to state that smaller cakes contain more cell wall material per surface area which possibly leads to harder cake crumb. From these results, it follows that egg yolk lipids are important for crumb softness. Graham et al. [18] suggested that lipids released from disintegrated low-density lipoproteins during baking lead to soft cakes as a result of being dispersed in the matrix and/or being adsorbed onto the protein–starch network. Low-density lipoproteins probably disintegrate during cake baking because apoproteins of low-density lipoproteins become part of the protein network [20,35]. This in turn decreases the degree to which apoproteins and phospholipids interact in the outer layer of the lipoprotein structure and eventually leads to lipoprotein disintegration. In studies in which different plasma-to-granule ratios were applied in the recipes of muffins [21,22] and Madeira cakes [38] egg yolk plasma was also found to contribute to product softness. Since egg yolk plasma contains 93% of all egg yolk lipids [5], these insights are probably also valid in the context of the present study even though muffins and Madeira cakes are completely different cake systems.

### 3.4. Hexane-Extractable Lipid Contents of Batter and Cake Samples

Above, we hypothesized that the solvent treatment resulted in low-density lipoprotein disintegration in EY_FD_ (see Section 3.2). To test this hypothesis the levels of lipids extractable with hexane from batter and cake samples (see Section 2.6) were determined. Lipids of intact low-density lipoproteins are not extractable with hexane, while those of disintegrated lipoproteins can be extracted using hexane.

A first finding was that the hexane extractable lipid content of EW_F_/EY_F_ cake was much higher than that of the corresponding batter (Table 3). This was because apoproteins take part in the mixed protein network during cake baking [20,35]. This likely disturbs apoprotein–phospholipid interactions leading to disintegration of low-density lipoproteins. At the same time, their nonpolar lipid core is released rendering the lipids extractable with hexane [18].

Hexane treatment of freeze-dried sponge cake (batter) samples extracts egg yolk lipids as well as some flour lipids. However, hexane extracts from batter and cake mainly contain egg yolk lipids since flour and egg yolk lipids make up ca. 1.2% and ca. 3.6% of batter dry matter, respectively. Only ca. 35% of the former (i.e., free flour lipids) are said to be extractable from the batter with hexane at room temperature [39]. Hexane extracts from cake may also contain internal starch lipids (i.e., flour lipids) that are released from the starch granules during baking as long as these lipids do not form amylose-lipid complexes during cooling [40,41,42].

Evidently, use of **EW_FD_/EY_F_** for sponge cake making rather than EW_F_/EY_F_ impacted neither the amount of lipids extractable from batter nor that from cake (Table 3). That the contents of hexane-extractable lipids of **EW_F_/EY_FD_** and **EW_FD_/EY_FD_** batters were only slightly higher than those of the EW_F_/EY_F_ batter (Table 3) indicated that freezing, drying and grinding did not severely impact low-density lipoprotein integrity. Hence, options on how these processing steps negatively impacted egg yolk aeration properties proposed in Section 3.1. are still plausible. Irrespective of whether EW_FD_, EY_FD_ or both were used for sponge cake making instead of their fresh counterparts, the levels of hexane-extractable lipids in the resulting cakes seemed similar (Table 3).

When egg yolk was not only freeze-dried and ground but also submitted to an ethanol treatment, the latter clearly disintegrated the low-density lipoproteins as the hexane-extractable lipid content of **EW_FD_/EY_FD,RL_** batter was significantly higher than that of EW_FD_/EY_FD_ batter (Table 3). The presence of a relocated or released egg yolk lipid fraction then likely interfered with the formation of a coherent protein layer at the air–liquid interface in the batter. As a result, gas cell stability decreased and EW_FD_/EY_FD,RL_ batter had a higher density than EW_FD_/EY_FD_ batter (see Section 3.2). Furthermore, it seemed that not all low-density lipoproteins had been disintegrated as a result of the ethanol treatment since EW_FD_/EY_FD,RL_ cake contained more hexane-extractable lipids than its batter (Table 3). Because less lipids were extracted with hexane from EW_FD_/EY_FD_ cake than from EW_FD_/EY_FD,RL_ cake, it also follows that EW_FD_/EY_FD_ cakes still contained intact low-density lipoproteins.

When egg yolk lipids had been removed prior to sponge cake batter preparation (**EW_FD_/EY_FD,D_**) hardly any lipids could be extracted with hexane from the resulting batter and cake (Table 3). These results thus confirmed what was stated above. Lipids extracted with hexane from batter or cake mainly originate from egg yolk.

The above results allow the hypothesis to be put forward that two low-density lipoprotein populations are present in sponge cake batter prepared with EW_FD_/EY_FD_ and three such populations in the resultant sponge cake. Since hexane-extractable lipid contents of batter and cake prepared with EW_FD_/EY_FD_ were similar to those of batter and cake prepared with EW_F_/EY_F_ (Table 3), it is very likely that this is also the case for conventionally prepared sponge cake (batter) (i.e., EW_F_/EY_F_ batter and cake).

**Sponge cake batter** presumably contains an intact low-density lipoprotein population (population I) which is dispersed in the aqueous phase of the batter and a damaged or disintegrated one at the air–liquid interface (population II). As about 0.5% of the batter dry matter consisted of hexane-extractable lipids that mainly originated from egg yolk, part of the low-density lipoproteins in batter must have been disintegrated. We hypothesize that population II adsorbed at the air–liquid interface where it disintegrated and released its components. This in turn rendered its lipids extractable with hexane.

**Sponge cake** presumably contained three low-density lipoprotein populations: (i) an intact low-density lipoprotein population (population I), (ii) a disintegrated one residing at the air–crumb interface (population II), and (iii) a second disintegrated population of which the apoproteins were incorporated in the protein network (population III).

The presence of population I in EW_F_/EY_F_ cake is suggested since such cakes did not contain the highest hexane-extractable lipid content observed (Table 3). The presence of such intact population has earlier been suggested by Graham et al. [18]. We further hypothesize that disintegrated lipoproteins at the air–liquid interface in sponge cake batter are retained during baking and cooling. In this view, population II is present at the air–crumb interface in sponge cake. Of relevance here is that X-ray photoelectron spectroscopy measurements have shown that higher levels of egg yolk lipids occur at the air–crumb interface than in the bulk of cake crumb [43]. Lastly, population III is disintegrated because of the incorporation of its apoproteins in the protein network [20,35]. For recipes containing egg yolk lipids, this was evidenced by the higher hexane-extractable lipid content measured for cake than for the corresponding batter (Table 3).

### 3.5. Starch Gelatinization and Protein Polymerization during Sponge Cake Baking

**Figure 3:**
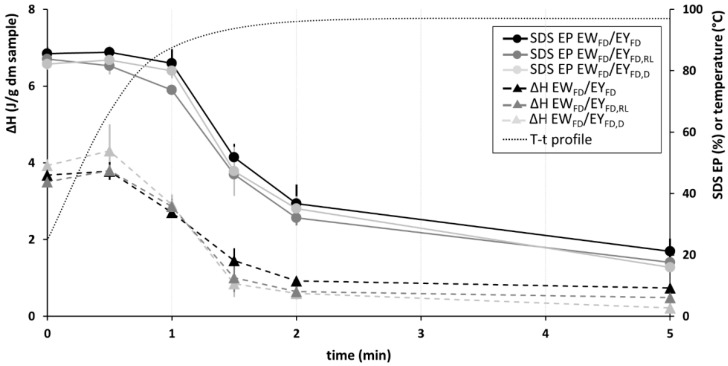
Enthalpy (ΔH, expressed in J/g dry matter (dm)) of the endothermic transition during differential scanning calorimetric (DSC) analyses and the percentage of protein extractable in sodium dodecyl sulfate containing medium (SDS EP) of heated + defatted sponge cake batter samples prepared from recipes containing different egg yolk treatments prior to batter preparation. Sample codes are shown in Table 1. For heating times exceeding 5 min, parameters remained constant. Vertical bars indicate standard deviations. Temperature-time (T-t) profile was measured using the Datapaq setup.

SDS EP and enthalpy values for heated + defatted batter samples prepared from sponge cake recipes containing EW_FD_/EY_FD_, EW_FD_/EY_FD,RL_ or EW_FD_/EY_FD,D_ are presented in Figure 3.

Enthalpy values of all batter samples first decreased between 30 and 60 s of heating in a water bath at 100 °C (i.e., sample temperature of respectively 67 and 88 °C) (Figure 3). Starch gelatinization and/or protein denaturation in EW_FD_/EY_FD,RL_ and EW_FD_/EY_FD,D_ were complete after 2 min of heating at 100 °C (i.e., sample temperature 96 °C) (Figure 3).

When the onset temperature of starch gelatinization and/or protein denaturation was determined in situ by heating the batter in a DSC device (i.e., online measurements, see Section 2.7), similar results were obtained for sponge cake recipes containing egg yolk subjected to the different treatments. Onset temperatures of the endothermic transitions were 88.5 ± 0.9, 88.4 ± 1.2 and 89.0 ± 1.1 °C for EW_FD_/EY_FD_, EW_FD_/EY_FD,RL_ and EW_FD_/EY_FD,D_ batters, respectively.

Based on the online and offline DSC experiments, we noted 85 to 90 °C as the temperature range over which starch gelatinization and/or protein denaturation started during sponge cake baking irrespective of whether egg yolk was solvent treated or not. These results are in line with earlier findings for sponge cake batter prepared with fresh eggs [27].

SDS EP levels and DSC enthalpy values decreased in the same temperature range (Figure 3) indicating that protein network formation and starch gelatinization and/or protein denaturation coincided during baking. Together, these phenomena set the cake structure and hence determined cake quality [19]. For all samples, protein polymerization continued during the isothermal phase of the heating process until constant SDS EP values were reached (i.e., after 5 min of heating at 100 °C).

Overall, prior solvent treatment of egg yolk did not clearly impact changes in SDS EP values during baking. This was in contrast to what was observed during boiling of egg noodles. When preparing noodles with hexane-defatted egg yolk, proteins polymerized faster during boiling than when EY_F_ was used [44]. This has been suggested to be caused by increased levels of hydrophobic protein–protein interactions in the absence of egg yolk lipids. Because of that, the protein network in noodles prepared with hexane-defatted egg yolk is believed to have a higher stiffness and strength than that in noodles prepared with EY_F_ [44]. Hence, while we here had expected faster protein polymerization during heating of EW_FD_/EY_FD,D_ batter than during that of EW_FD_/EY_FD_ or EW_FD_/EY_FD,RL_ batter, the obtained SDS EP values did not support this view.

Based on the above data, we conclude that relocation/release or removal of egg yolk lipids prior to batter preparation had no significant impact on the time and temperature dependence of starch gelatinization and/or protein denaturation and protein polymerization. These results could therefore not be related to the much harder texture of EW_FD_/EY_FD,D_ cakes (see Section 3.3).

## 4. Conclusions

In this study, the role of egg yolk lipids during sponge cake making was investigated by altering their location or removing them prior to batter preparation using ethanol treatments. The impact of using treated egg yolk samples on sponge cake (batter) properties and cake structure setting (i.e., starch gelatinization and protein polymerization) was evaluated.

The results clearly demonstrated that egg yolk lipids and more specifically the lipoprotein structures in which they occur are important for producing high-quality sponge cakes. This is the first study to show that egg yolk lipids determine sponge cake quality by virtue of their surface-activity properties at the air–liquid interface in the batter and not by affecting timing and/or the extent of cake structure setting during late baking. To prepare sponge cakes using a multistage mixing procedure preferably intact, low-density lipoproteins or, alternatively, their separate components are needed to incorporate enough air during mixing and stabilize it after mixing and during early baking. Only then cakes of high volume and soft texture will be obtained.

In addition, clear evidence for the existence of different low-density lipoprotein populations in sponge cake (batter) was provided. Populations were assigned based on lipoprotein integrity and location in the batter or cake. Batter contains two lipoprotein populations: (i) an intact one dispersed in the aqueous phase of the batter, and (ii) a disintegrated one at the air–liquid interface. We further suggest that sponge cake contains three low-density lipoprotein populations: (i) an intact one dispersed in the cake crumb, (ii) a disintegrated one residing at the air–crumb interface, and (iii) a second disintegrated one of which the apoproteins are incorporated into the protein network.

## Figures and Tables

**Table 1:** Sample codes of sponge cake (batter) prepared with different egg white and egg yolk treatments prior to batter preparation. Egg white was either fresh or freeze-dried. Egg yolk was fresh, freeze-dried or freeze-dried and treated with a solvent (egg yolk with relocated lipids or defatted egg yolk).

Sample		Sample Code	Number of Batters Prepared
Egg White	Egg Yolk		
Fresh	Fresh	EW_F_/EY_F_	1
Freeze-dried	Fresh	EW_FD_/EY_F_	1
Fresh	Freeze-dried	EW_F_/EY_FD_	1
Freeze-dried	Freeze-dried	EW_FD_/EY_FD_	3
Freeze-dried	With relocated lipids	EW_FD_/EY_FD,RL_	3
Freeze-dried	Defatted	EW_FD_/EY_FD,D_	3

**Table 2:** Properties of sponge cake batters and sponge cakes prepared with different egg white and/or egg yolk treatments prior to batter preparation. Sample codes are shown in Table 1. Standard deviations are indicated between brackets.

Sample	Batter Viscosity (Pa*s)	Batter Density (g/mL)	Cake Volume (mL)	Corrected Hardness (N*cm^3^/g)	Cohesivenes (-)	Springiness (-)	Average Cell Size (mm^2^)	Number of Cells/cm^2^
EW_F_/EY_F_	n.m.	0.68 (0.00)	386 (4)	8.6 (0.4)	0.73 (0.01)	0.89 (0.00)	0.36 (0.02)	83 (4)
EW_FD_/EY_F_	n.m.	0.68 (0.01)	378 (1)	8.6 (0.5)	0.72 (0.01)	0.88 (0.01)	0.36 (0.03)	80 (3)
EW_F_/EY_FD_	n.m.	0.75 (0.01)	352 (1)	9.7 (0.6)	0.73 (0.00)	0.89 (0.01)	0.31 (0.02)	82 (6)
EW_FD_/EY_FD_	138 (10) ^b^	0.75 (0.01) ^b^	355 (13) ^a^	9.0 (0.8) ^b^	0.72 (0.01) ^a^	0.89 (0.00) ^a^	0.30 (0.02) ^b^	94 (8) ^a^
EW_FD_/EY_FD,RL_	158 (11) ^a^	0.88 (0.02) ^a^	293 (8) ^b^	8.5 (0.8) ^b^	0.68 (0.02) ^c^	0.87 (0.01) ^b^	0.35 (0.03) ^a^	84 (9) ^b^
EW_FD_/EY_FD,D_	170 (11) ^a^	0.89 (0.03) ^a^	254 (9) ^c^	19.5 (2.0) ^a^	0.71 (0.01) ^b^	0.89 (0.01) ^a^	0.37 (0.03) ^a^	84 (4) ^b^

Results in the same column with different letters are significantly different (α < 0.05). Batter and cake properties resulting from sponge cake recipes that were processed more than once were statistically analyzed. n.m.: not measured.

**Table 3:** Hexane-extractable lipid content (expressed as a % of sample dry matter (dm)) of sponge cake (batter) samples prepared from recipes containing different egg white and/or egg yolk treatments prior to batter preparation. Sample codes are shown in Table 1. Standard deviations are indicated between brackets.

Sample	Hexane-Extractable Lipid Content (% of dm)	
	Batter	Cake
EW_F_/EY_F_	0.34 (0.14)	2.43 (0.19)
EW_FD_/EY_F_	0.49 (0.14)	2.77 (0.22)
EW_F_/EY_FD_	0.87 (0.20)	2.37 (0.05)
EW_FD_/EY_FD_	0.58 (0.20) ^b^	2.53 (0.19) ^b^
EW_FD_/EY_FD,RL_	2.34 (0.57) ^a^	3.77 (0.21) ^a^
EW_FD_/EY_FD,D_	0.05 (0.12) ^c^	−0.03 (0.33) ^c^

Hexane-extractable lipid contents of batters and cakes prepared from sponge cake recipes that were processed more than once were statistically analyzed. Results in the same column with different letters are significantly different (α < 0.05). Standard deviations of hexane-extractable lipid contents of batters and cakes prepared from sponge cake recipes that were processed once (i.e., top three rows) represent the deviation within one batch of cakes. Standard deviations of hexane-extractable lipid contents of batters and cakes prepared from sponge cake recipes that were processed more than once (i.e., bottom three rows) include the deviation introduced by the different treatments and by the preparation of multiple cake batters.

## Data Availability

Data available on request.
